# Controlling Chaos: The Perceptions of Long-Term Crack Cocaine Users in Vancouver, British Columbia, Canada

**DOI:** 10.1155/2013/851840

**Published:** 2013-07-24

**Authors:** Steven Persaud, Despina Tzemis, Margot Kuo, Vicky Bungay, Jane A. Buxton

**Affiliations:** ^1^Communicable Disease Prevention and Control, British Columbia Centre for Disease Control, 655 West 12th Avenue, Vancouver, BC, Canada V5Z 4R4; ^2^School of Population and Public Health, University of British Columbia, 2206 East Mall, Vancouver, BC, Canada V6T 1Z3; ^3^School of Nursing, University of British Columbia, 302-6190 Agronomy Road, Vancouver, BC, Canada V6T 1Z3

## Abstract

People who smoke crack cocaine are described as chaotic and more likely to engage in risky sex, polysubstance use and contract infectious diseases. However, little is known about how individuals perceive smoking crack as compared to other forms of cocaine use, especially injection. We explored the lived experience of people who smoke crack cocaine. Six gender-specific focus groups (*n* = 31) of individuals who currently smoke crack in Vancouver, Canada, were conducted using a semi-structured interview guide. Focus groups were transcribed and analyzed by constant comparative methodology. We applied Rhodes' risk environment to the phenomenological understanding that individuals have regarding how crack has affected their lives. Subjects reported that smoking rather than injecting cocaine allows them to begin “controlling chaos” in their lives. Controlling chaos was self-defined using nontraditional measures such as the ability to maintain day-to-day commitments and housing stability. The phenomenological lens of smoking crack instead of injecting cocaine “to control chaos” contributes a novel perspective to our understanding of the crack-smoking population. This study examines narratives which add to prior reports of the association of crack smoking and increased chaos and suggests that, for some, inhaled crack may represent efforts towards self-directed harm reduction.

## 1. Introduction

 Smoking crack cocaine is a relatively neglected public health problem in Canada in comparison to injection drug use (IDU), despite indications that crack use in Canada is increasing. The Vancouver Injection Drug Users Study found that crack use in a group of injection drug users in Vancouver almost doubled from about 31% in 1997 to over 60% in 2004; and daily crack smoking in this population rose from <10% in 1996 to 40% in 2002/05 period [1]. 

 Canadian data also provides evidence of high prevalence of crack use among drug user populations. A recent surveillance report of 794 people who inject drugs across Canada indicated that 52.2% of the total sample had also used crack cocaine in the last 6 months [2]. Another Canadian cohort of illicit opioid users in five cities indicated that 54.6% (371/679) of baseline participants had smoked crack in the 30 days prior to the survey. However, there was considerable regional variation, with crack use reported ranging from 86.2% in Vancouver to 2.4% in Quebec City [3]. On the other hand, in a study of people who smoked crack in Vancouver only 39% of those who smoked crack also reported injecting drugs [4]. In Canada, many services for people who use drugs are targeted to people who inject; therefore, people who only smoke crack may not be linked with appropriate health and social services.

 Compared to other drug using populations, people who use crack cocaine are described as a particularly chaotic population. Crack using populations are more likely than nondrug using populations to engage in illegal activities, to experience homelessness and health problems; yet they are less likely to access health and social services [5]. While cocaine users in general are at elevated risk of risky sexual practices, the crack house environment has been implicated in increased “sex for crack” exchanges and unprotected sexual encounters [6, 7]. DeBeck et al. (2009) reported that crack smokers smoke a median of 4 times per day [1]. Results of a population-based study revealed that recent-onset crack cocaine smokers were about twice as likely to experience cocaine dependence, as compared to recent-onset cocaine HCl powder users who did not smoke crack [8]. Crack smoking also involves particular risks and harms, including HIV incidence [1], potential HCV and tuberculosis transmission, and agranulocytosis from crack cocaine containing levamisole [9–14].

 Vancouver's downtown eastside (DTES) has a longstanding reputation as Canada's poorest urban area. Through a historical trajectory of diminishing housing options, an increase in single-room occupancy rentals and a mass influx of marginalized individuals have led to a disproportionate concentration of individuals with mental illness, addiction, and an HIV/AIDS epidemic termed by the local health board as a “public health emergency” [15, 16]. 

 Although harm reduction interventions such as needle/syringe distribution and a supervised injection site are available in the DTES neighborhood, the majority of evidence-based public health responses to problematic drug use in British Columbia (BC) are not tailored for people who smoke crack cocaine and their unique needs. Plastic mouthpieces, push sticks, and, recently, brass screens are provided by the provincial harm reduction program. However, glass stems are not currently available through this program [17].

 Crack cocaine use is a growing concern in Canada with many associated harms. Yet, there is no clear understanding of the perception of harms related to the type of cocaine (powder cocaine versus crack cocaine) and mode of administration (snorting, smoking, and injecting) by people who use crack. The psychosocial determinants and factors related to crack cocaine initiation, continuation, and the trajectory of substance use by mode of administration have not been well identified either. To assist in developing appropriate services there is an urgent need to learn more through a qualitative study of people who smoke crack. 

 To this end, this study sought to investigate the lived experience of crack smokers, especially in comparison to injected cocaine and other drugs in the context of the DTES. This contributes to our still limited understanding of this population with the ultimate aim to provide health service providers and policy makers with information to better design, plan, and implement prevention programs at an individual and community level.

This study was informed by two theoretical frameworks: phenomenology and Rhodes' risk environment. The phenomenological framework elucidates data from participants, treating their subjective perceptions as real insofar as it shapes their worldview and behaviors. This individual understanding of events and behaviors is significant in interpreting the meaning that people attribute to various things, and therefore, why they respond in certain ways. It “seeks to understand the lived experiences of individuals and their intentions within their “life world”” [18].

 The risk environment framework, first applied by Rhodes (2005) to explain the factors affecting HIV risk, describes the “space, whether social or physical in which a variety of factors exogenous to the individual interact to increase vulnerability” [19]. This holistic understanding of the complex interplay of what Zinberg terms “set and setting” [20] or intrinsic attitudes and structural factors that contribute to overall risk is useful in identifying potential areas of harm reduction. 

## 2. Methods

 A semi-structured interview guide was developed in collaboration with stakeholders including local service agencies and people who use drugs. Due to the overwhelming response usually seen with general recruitment strategies such as fliers, participants were purposively selected, based on ability to meet study criteria and lived experience, by the 2 organizations located in the Vancouver DTES neighborhood. One organization is a peer-support agency for people who use drugs, and another is a shelter and multiservice centre for women in survival sex work. Stakeholders at each of these agencies were instructed to approach potential participants with informational material and contact information of the study investigators if they chose to participate. Participants were 19 years old or older, able to speak and understand English, smoke crack at least 3 times per week in the past year, and provided written consent for participation prior to focus groups. Ethics approval was received from the University of British Columbia Behavioral Ethics Board, and standard ethical research practices, including voluntary consent, confidentiality, and minimal risk of harm to participants, were maintained.

### 2.1. Focus Groups

 In total, 10 male and 21 female participants were recruited, and 6 semi-structured focus groups were conducted (2 male, 4 female), each comprised of 5-6 participants and 2 coinvestigators. One coinvestigator facilitated discussion, and the other took field notes. Focus groups were conducted at the respective recruiting agencies between June and November 2011. These focus groups were 1 hour long and were recorded and transcribed for data analysis. A $20 honorarium was given to participants. The use of gender-specific focus groups allowed for frank discussion between participants, as both men and women were able to discuss gender norms openly and comment on interactions with the opposite gender with relative safety. In discussing topics with other crack users, participants were also able to highlight both areas of consensus and norms, as well as individual opinions, providing a distinction which would have been difficult using individual interviews. 

### 2.2. Data Analysis

 Transcripts were cleaned of any identifying data and were loaded into qualitative analysis software QSR NVivo 8 [21]. They were then reviewed by the two coinvestigators facilitating the focus groups for content and orientation. An initial line-by-line free coding was conducted using a constant comparative approach, in keeping with grounded theory research [22]. Themes and conceptual categories were identified and constantly compared to each other in order to develop codes, which represented both areas of commonalities between and within groups, as well as unique or opposing information.

Themes and impressions were then discussed between researchers, and an axial coding was then conducted for more focused themes and more insight into findings. This process involved a higher level of coding which compares and organizes themes according to a theoretical framework to better conceptualize their relative significance, hierarchy, or relation to established theory. In our case, Rhodes' risk environment was used to describe codes relating to the various perceptions of risk, and a phenomenological lens explained what role smoking crack represented in participants' lives. Member checking was conducted in the form of poster presentations at the same agencies, confirming central themes. Member checking included 5 participants who took part in the focus groups and 12 other individuals who were users of the service.

## 3. Results

 Demographic information of study participants is found in Table 1. Though the proportion of aboriginal participants is quite high, this is representative of the DTES population, which includes between 10 and 14% aboriginal individuals, [15] compared with the national average of 3.8% [23]. The two theoretical frameworks yielded a number of themes through two conceptual ideas: the phenomenological notion of controlling chaos and Rhodes' risk environment. The first sought to identify participants' own perceptions regarding smoking crack, what it meant to them and how it affected their daily lived experience. The latter described the social, economic, and physical risks unique to smoking crack. Findings presented here represent only those themes with strong consensus and little to no dissent from both within and between the different focus groups, as identified through transcripts, field observations of nonverbal cues, and comparative analysis.

### 3.1. Phenomenological Lens: Controlling Chaos

 In the midst of an admittedly chaotic lived experience, smoking crack represented both internal and practical efforts by users to exert control and autonomy over their drug use and other aspects of daily life. It effectively represented them “controlling chaos”, as manifested in examples of the subjective experience, means of reducing harm, and reprioritizing drug use within the context of other daily needs. Participants constantly referred to the chaos that previously defined their lives, and how they were better able to control it now that they smoked rather than injected.

*Female Participant: we're past the part where we're in the chaos *… *I'm not living the same lifestyle anymore.*

### 3.2. Subjective Experience

 Many participants viewed crack cocaine use as improvements in their stability and control. This theme was confirmed in 5 of 6 focus groups:

*Male Participant: So this [crack] sort of keeps me, you know, keeps me away from doing anything worse that I could do. *

*Female Participant: I'm a functioning user. I don't miss things because of dope [crack]. I still have to pay my rent *… *dope comes last.*

One of the most explicit examples of controlling chaos came from the subjective high of the drug itself. Smoked crack was described as more gradual in onset, especially when compared to the instantaneous rush of IV cocaine use. Similarly, the sustained drug effect is much more vivid when injecting than smoking.

*Female Participant: Oh, an injection hits you right away, it's—it gives you the—wham!*

*Female Participant: To the cliff's edge right now. *

It is important to note that unlike a consistently intense experience with injecting, the high of smoking crack was widely variable and highly dependent on the agents added, either to add inert bulk to the drug (wax) or as an adulterant with additional effects (levamisole [13], methamphetamine). In this way, users give up a measure of certainty of their high. However, the delayed onset makes it easier to temper some of the impulsive and risky actions, including high-risk sexual activity that is often associated with drug use.

While some participants described feeling less paranoia than with injecting, particularly with respect to police interaction, others were aware of more “tweaking” and other side-effects of the additives. Many users said that they would still prefer the high and subjective experience of injecting and choose to smoke for other reasons. For many, it is due to the perceived improvements in overall stability and control.

*Male Participant: when I compare injecting to smoking the pipe, injecting was more intense, more expensive, more time consuming. It was always on my mind. It was harder for me to shut it down out of my mind when I was injecting … I just finally decided that I had more control over the drug by smoking. *

*Male Participant: when I do inject, yeah, everything's just—spur of the moment.*

### 3.3. Drug Use Trajectory

 Participants were also able to contextualize crack smoking with respect to other drugs. The majority smoke crack either in addition to injecting cocaine or as a replacement for this. For many, this change was out of necessity as they could no longer find viable injection sites or fear of contracting infections after having contracted HCV from injection. Others were introduced by a partner or spouse, and many decided that it was safer or associated with less stigma. Nevertheless, delineating a common trajectory of drug use is difficult. General consensus among study participants was that many new users start with inhalation without ever injecting, and one's drug route of choice is highly individualistic.

*Female Participant: I think everybody's different … Anything starts any way, it's 2011, anything goes now.*

### 3.4. Polysubstance Use

 The significance of polysubstance use can be explained within the phenomenological framework of controlling chaos. In an intentional way of attempting to reduce drug use, several participants reported using beer or marijuana to stave off cravings between highs. This allows them to go longer without smoking crack and to reduce their overall use.

*Male Participant: And that's where the pot comes into play because that replaces—takes away the craving of me wanting more. So the pot helps me to be able to maintain when I can't afford any more crack*

*Female Participant: That's what I do. I use a little bit of weed with my crack … yeah, it kind of helps me cope with the—crave the—you know*

*Female Participant: I find that I usually smoke less rock if I have the weed [be]cause it helps with the jonesing or takes the crave down.*

### 3.5. Reprioritizing

 Injection use is described as an ever-present concern, where users are constantly thinking about their next high. Conversely, smoking has allowed some users enough reprieve to focus on other pursuits without the constant and pervasive craving. In this way, the role of crack in the daily lives of users seems to allow for more stability. While using IV drugs, participants reported their drug use taking priority over basic needs such as shelter and food. In contrast, smoking crack seemed to be lower on their list of priorities, often coming second to housing, food, or family relationships. This enabled participants to maintain obligations to employers, relationships with family and friends, and self-care and finances issues which were frequently and spontaneously raised when participants described their level of stability or chaos.

*Female Participant: My bills are all paid. I don't owe money. I don't do crime anymore. I keep my appointments, you know, all stuff. So it's like people going out and having a glass of wine at lunchtime and bringing home a six-pack or something. I like to take home—when I go home in the evening, I like to take a little—like, 20 piece or something home and then watch TV.*

*Female Participant: I still do all my jobs that—my little volunteer stuff that I'm supposed to do-*

*Male Participant: for me it is a lot easier to accomplish cutting myself off the rock for a substantial amount of time so that I am eating proper, sleeping proper and taking better care of myself. And it's given me back a lot of my self-respect and respect for others, too.*

### 3.6. Risk Environment

 The risks of crack smoking are multifactorial and involve characteristics of the drug itself, the social environment, especially as it pertains to gender inequalities, and the physical elements of place. 

### 3.7. Infection and Safety

 Though many acknowledged an understanding that infection is still a reality, participants readily agreed that smoking is a safer way of consuming drugs than injecting. Frequent anecdotes of cellulitis, phlebitis, and other soft tissue infections, as well as HCV transmission with previous IDU, cause many participants to assert that smoking is the safer alternative. In addition, the gradual onset of smoking drugs could help reduce overdose events; most respondents maintain that their risk of overdose is much less than when they inject.

*Male Participant: Injecting is everything … I seen some really bad infections that opened, like, you know, where you could see inside the arm …*

Despite a fundamental knowledge of how disease is spread through smoking crack, participants still reported sharing crack pipes, doing so much more frequently than they would with needles.

*Male Participant: Pipes are shared a lot.*

*Female Participant: People know—can I borrow your pipe, can I borrow your pipe? And … gives them a toke up. *

### 3.8. Quality

 Some of the biggest health concerns relate to cutting agents and uncertain drug quality, as well as the respiratory complications of inhaling Brillo, used as a screen to avoid inhaling crack. Moreover, the wide range of quality and variation in cutting agents mean that there is rarely any certainty about what and how much one is smoking in any given rock. Many, though not all, focus group participants were aware of levamisole being used as a cutting agent and the health effects of agranulocytosis that may result, accepting this and other cutting agents as a dangerous but unavoidable consequence of the variable quality of crack. The same is true of adulterants such as methamphetamine which, when added, may cause tweaking, paranoia, and unpredictable behavior.

*Male Participant: when it first came out, like, when you knew rock was fucking rock, it was real. And you used to get high. Now it's just fucking pills and baking soda.*

### 3.9. Social Environment

Though crack pipes are more likely to be shared, most describe the high as being an antisocial experience and say that they are more likely to smoke alone or seek solitude when smoking crack, worried that someone will ruin their high, interrupt the experience, or judge them for tweaking.

*Male Participant: it's not a social event when you're getting high … it's a selfish drug, isn't it.*

*Female Participant: it tends to make me isolate even more, because I'm in there and it's like to leave is, like, a—that's a major, major feat.*

### 3.10. Gender and Sex

 Crack users do not associate smoking with an increase in risky behaviours as compared to injected cocaine, and in fact, associate injecting with more spontaneity. Specifically with respect to risky sexual practices, participants believe that smoking crack is an inhibitor of spontaneous sex, both genders agreeing that most men cannot achieve an erection and consider it a futile endeavor.

*Female Participant: They can't get it up. I'm saying, it's just too much … when I'm doing the crack, I do not even want the sex.*

However, despite the clearly unproductive effects of crack on sexual activity, some women also describe men wanting to initiate sexual encounters. Interestingly, although male users claim that they rarely attempt sexual encounters, female users highlighted it as a frequent request and shared anecdotes of crack-for-sex transactions.

*Female Participant: A lot of the guys that I smoked with, they always wanted something in return sexually.*

There is a highly gendered element to the use of smoked crack, especially evident in the stories of how participants began smoking crack. Men often describe a highly individualistic choice weighing factors such as cost, availability, inability to find injectable veins, and fear of infection.

*Male Participant: If I had veins now I'd be doing powder again. So this sort of keeps me, you know, keeps me away from doing anything worse that I could do.*

Conversely, the vast majority of women began smoking crack or switched from injecting to smoking either with a male partner or family member or at the request of a partner or spouse.

*Female Participant: I moved to Vancouver and started smoking crack [be]cause my spouse at the time didn't want me fixing down here [be]cause he thought maybe I might O.D. … so he made me start smoking crack. *

The contrasting experiences of different genders as well as the further marginalization of female crack smokers [24] are seen in one male participant's comments of sharing crack as a means of achieving power and companionship.

*Male Participant: I like sharing with the opposite sex. Gets me that power, what you really want to call it, because you feel like you got something that somebody wants … because of my age [60 years], the un-security of saying okay,… you're wanted.*

### 3.11. Housing and Place

A common theme highlighted by study participants was the need for a safe place. Individuals unanimously agree that given the choice, they would much rather smoke inside as they are less likely to be assaulted or have a high ruined by paranoia. The ability to smoke crack in one's own home leads to a more controlled, safer experience. Several anecdotes outline altercations in crack houses where a user became violent with other occupants, they were harassed by police, or of vulnerability to street crime.

*Female Participant: And if you don't want us smoking in our safe haven, which was looking out for us and us looking out for each other … you're putting me and my sisters looking out in the alleyways at 3:00 in the morning, for a safe place to—which leads us, you know, to guys raping us, to robbing us, to getting us set up. Just because the neighbourhood wants to feel better. *

*Female Participant: … especially women. But it also happens to guys. They're out in the back alley and they're getting robbed, getting punched out … if you're in a place, a safe place, you have people around, that's not going to happen.*

*Female Participant: if it's legalized [inhalation room], you know, you do not have to worry.*

Safe housing has additional significance to participants as both a sign of their increasing stability, as well as a protective factor in the control and moderation that they seek to achieve over their drug use. The following quote illustrates a belief that was reiterated by almost all participants; stable housing contributes to increased stability in life and drug use.

*Male Participant: I have a place to live now for a little over two years and that makes a tremendous difference on my usage. I'm able to keep more of a handle on my usage before it starts getting way out of control. And it—because I have a home now there's other things that are higher priority for me.*

Despite the highly reclusive experience of crack smoking, some participants identified a supervised inhalation site as the most helpful strategy to assist with harm reduction. This was widely supported by all participants, with no participant disagreeing that this would be a beneficial strategy. This location would give users an indoor location to smoke, safe from harassment and police interference often experienced on the street, as well as worry about being disturbed. As well, they would have access to resources for health education and other social resources.

*Male Participant: [a] safe injection site but for smoking crack … somewhat social, but also to learn, right, to learn more of the addiction and to learn—and remind myself that there are caring people who care and that there ways out, you know. Because sometimes I do forget and I feel that I'm trapped in this addiction forever, right.*

## 4. Discussion

 This study represents a novel application of two theoretical frameworks to illuminate the experiences of those who use crack. Drug use has been well studied; however, while a great deal of the literature assumes a measure of equivalence between injection and inhalation, we have found crack inhalation to be notably different from other methods of cocaine consumption. 

### 4.1. Controlling Chaos

 The meaning that individuals who smoke crack attribute to their drug use adds an important voice to the discourse surrounding crack smoking. Studies such as Hatsukami and Fischman [25] describe crack users as leading especially chaotic lives and being at increased risk of engaging in unsafe sexual practices, polysubstance use, and contracting HIV or HCV. Our findings suggest that our participants view it in a different light. They report qualities of smoked crack that allow them to mitigate this chaos, including the subjective experience, and addictive potential. 

 Our findings both confirm and challenge the current understanding of increased polysubstance use. While they confirm previous reports of a higher likelihood of polysubstance use, [26] our findings suggest an alternative interpretation of the significance of this. Instead of representing more chaos in the crack smoker's life, our study participants describe their use of these other drugs as an intentional effort to reduce their crack consumption. Notably, our sample was comprised of a number of older individuals, many of whom had prior injection drug use and had transitioned to crack smoking. While these findings may not hold true in less seasoned drug users or with respect to other drug use trajectories, this population described a novel understanding and perception of crack which should be at least considered when attempting to explain the significance of these often observed behaviors. Instead of a measure of chaos, polysubstance use is a means of decreasing frequency of crack use—effectively, a harm reduction strategy. While many studies have confirmed this polysubstance use, very few seek to explain its meaning, and while the assumption of increasing chaos may be largely true, this study proposes an alternative view based on the beliefs and perceptions of the participants themselves.

 This phenomenological contextualization of smoking crack certainly leads to unexpected conclusions regarding its subjective meaning, but even more significant are its implications for the greater discourse on smoking crack. Much of our understanding of the instability of smoking crack is evaluated through metrics such as HIV and HCV transmissions, risky sexual practices, polysubstance use, and dependence on cocaine. Throughout the transcripts, however, efforts to elucidate participants' level of stability were constantly met with declarations of bills paid on time, relationships and obligations maintained, and permanent housing and employment sustained. Thus, when asked to self-report their control, individuals who smoke crack used not only a different set of metrics to measure this but also a fundamentally different dimension. 

 Our understanding of the chaos or control experienced by crack users may be ignoring important qualitative measures of maintaining obligations and social roles. Previous qualitative studies support our findings that an important self-identified measure of whether an individual is a stable or unstable user relates largely to subjective priority-setting and interrelational factors [27]. By reframing our notion of control through these self-identified metrics, we gain a more holistic and accurate picture of the lived experience of individuals who smoke crack.

### 4.2. Risk Environment

 As speculated and previously observed, the social, political, economic, and physical risk environments within which individuals who smoke crack reside are rife with potential harms, many of which are singular to crack use. 

Despite its favorable comparison to IDU, the real threat of infectious disease transmission is not overlooked by participants. Nevertheless, they still report sharing crack pipes, a clear route of transmission of disease [1, 28]. This mirrors previously observed trends, where one estimate was that over 47% of Vancouver crack smokers reported sharing a crack pipe in the previous 6 months [28]. Most of the attention surrounding crack use has been in relation to the transmission of HCV and HIV. Clearly, this is a serious risk, both due to the disproportionate prevalence in this population, and the persistence of HCV on crack pipes and oral sores common to crack smokers [9]. There are a number of potential reasons for sharing pipes: it may be a product of the greater drug culture and the norms surrounding sharing of drugs, which may be supported by the insight that many new users were initiated into crack smoking by sharing a pipe with someone else. It may be a form of risk compensation, with the understood reduction in transmission risk emboldening individuals to share more readily. However, the most commonly repeated reason is related to the fear of crack pipes being confiscated and destroyed by police. The threat of police harassment, combined with the personal cost of crack pipes, may be creating a disincentive to carrying one's own pipe. As Leonard et al. demonstrated, increasing the accessibility of harm reduction supplies is effective in reducing the sharing of drug smoking paraphernalia [29], suggesting that a way of mitigating both the social risks of police harassment and physical risks of infection lies in facilitating harm reduction supply distribution with the cooperation of law enforcement. 

The implications of substantially variable strength and quality of crack are major concern. Much of the instability surrounding crack is blamed on the unpredictability of a given high. The addition of methamphetamine and other drug adulterants causes tweaking and paranoia, which adds an element of danger in communal use and may be an inciting factor in the desire for solitary consumption. While participants had no clear suggestion for how to mitigate this risk, identifying it is an important first step. Concerns about increasing risky sexual practices are not borne out in our findings. Women certainly describe trading sexual favors for the use of a crack house, drugs or materials, and as part of the experience of sharing drugs with a partner. Maranda et al. [30] highlight an association between increased sexual partners and crack use as justification for the folk belief that it increases libido. However, crack is clearly not portrayed in our findings as a sexually stimulating drug, and sexual activity may be related more to the gendered power differentials prevalent in this population. 

Certainly, the marginalization associated with smoking crack seems to disproportionately affect females. Focus group data seems to support a different trajectory into smoking crack, associated with more external influence and perhaps even coercion. Women recruited from an organization helping survival sex workers understandably had more to say on this matter; however, recruitment from the broader population of women who use crack also endorsed a sexual dimension very different than that of male participants. 

### 4.3. Housing and Place

Housing was noted to be an important factor in increasing the overall stability and control over users' addiction,= as well as mitigating many of the inherent dangers of smoking crack. The significance of safety and stability lends further support to the housing first philosophy that stable housing is a precursor to, and not a consequence of, addictions treatment [31]. Also, the strong support for a supervised inhalation site and access to resources from a group which, by their own admission prefer isolation, speaks to the benefit which they see in such a facility.

### 4.4. Harm Reduction Strategies

A number of harm reduction strategies were identified by participants, both directly and by virtue of the obstacles to safer inhalation. Most notably, a supervised inhalation site was ubiquitously supported. According to DeBeck et al., 71% of surveyed crack smokers would use such a facility, and it could serve as a distribution site for safe inhalation supplies and a means to connect people who smoke crack cocaine to health and social service providers [32]. Though men also suggested the benefit of a safe inhalation facility, women seemed especially emphatic when speaking of the risks surrounding the lack of safe space. Handlovsky et al. [33] also found housing and the concept of safe places to be a key determinant of harm-reduction in female crack smoking populations. 

Similarly, users described various strategies for reducing crack use, largely by mitigating cravings with alcohol and cannabis. Beyond the health effects of reducing crack use, the agency exercised in rationing crack may be empowering and encouraging. A significant risk characteristic in crack smoking is the uncertainty surrounding drug quality and cutting agents. Employing initiatives aimed at improving quality and consistency of crack cocaine would allow for a much more predictable high and reduce the risk of immune compromise from levamisole.

### 4.5. Limitations

 For logistical reasons, the sample of study participants was recruited from individuals already using the two support service organizations. This poses a potential bias, as users connected to peer support agencies may represent a subset of drug users who have greater access to resources or are at a specific place in terms of their drug use trajectory. This may yield insight into why there is an abundance of previous injection use amongst study participants despite the claim that there is no clear escalation from one method to the other and that many new users begin by smoking crack. Additionally, the median age of our sample population was 47 years and may reflect a longer drug use career, implying higher chance of damaged veins, maturity, and a specific trajectory that is not representative of the greater crack smoking population. Were it feasible, a broader demographic range may reveal disparate trends between newer cocaine users and those who have been using it for several decades.

## 5. Conclusion

 Smoking crack is, in fact, very different than other forms of cocaine in both subjective experience and impact on the lives of users. Employing a phenomenological framework yielded many insights into the lived experience of crack inhalation, but more importantly, contributed the qualitative dimension of meaning to previously documented observations regarding populations who smoke crack. Through this lens, we posit that for some, especially those with long-standing drug use careers, smoking crack may represent an increase in efforts to control chaos, and the observed polysubstance abuse is a mechanism for reducing overall crack consumption. The conclusions of this study are not intended to describe the entirety of crack using populations, but rather to highlight an alternative understanding that illustrates one of the many ways that some individuals view crack use as compared with injected cocaine and other drugs. 

 Moreover, the measures by which individuals define their own level of control or stability incorporate metrics which are often neglected by much of the literature when describing the same. By exploring the qualitative perceptions and understanding of observed trends in drug use, further studies can explore the meaning that people who use drugs attribute to their behaviors. 

 Additionally, the significant variability in drug quality, constant threat of violence, further marginalization of women, and lack of a safe place to get high all pose concerns from a health standpoint and offer opportunities to create relevant harm reduction initiatives to address them.

## Figures and Tables

**Table 1:** Demographic information of study participants*.

Variable	Totals (%) *N* = 31
Sex *n* (%)	
Female	21 (67.7)
Male	10 (32.3)
Age years	
Mean (range)	47.15 (27–64)
Ethnicity *n* (%)	
Caucasian	11 (35.5)
Aboriginal	19 (61.3)
Other	1 (3.2)
Duration of crack use years	
Mean (range)	10.4 (3–25)
Injection drug use *n* (%)	
Currently use	26 (83.9)
Ever used injection drugs	31 (100)

*Four focus groups were conducted at a support agency for people who use drugs (2 male, both *n* = 5; 2 female, both *n* = 5). Two focus groups were conducted at a multiservice centre for women in survival sex work (*n* = 5, *n* = 6).
